# Fibroblast Activation Protein Inhibitor (FAPI) PET: A Scoping Review of Emerging Oncologic and Fibroinflammatory Applications

**DOI:** 10.3390/diagnostics16101542

**Published:** 2026-05-19

**Authors:** Emmanouil Panagiotidis, Filippos Koinis, Jules Zhang-Yin, George Angelidis, Varvara Valotassiou, Ioannis Tsougos, Athanasios Kotsakis, Panagiotis Georgoulias

**Affiliations:** 1 Nuclear Medicine Laboratory, University Hospital of Larissa, 413 34 Larissa, Greece; 2 Department of Medical Oncology, University Hospital of Larissa, 413 34 Larissa, Greece; 3 Department of Nuclear Medicine, Clinique Sud Luxembourg, Vivalia, 6700 Arlon, Belgium; jules.zhangyin@vivalia.be; 4 Medical Physics Laboratory, University Hospital of Larissa, 413 34 Larissa, Greece

**Keywords:** fibroblast activation protein inhibitor (FAPI), cancer-associated fibroblasts (CAFs), PET/CT, theranostics, standardized imaging protocols, artificial intelligence, PRISMA-ScR

## Abstract

This scoping review was conducted in accordance with the Preferred Reporting Items for Systematic Reviews and Meta-Analyses Extension for Scoping Reviews (PRISMA-ScR) guidelines. It summarizes advances in fibroblast activation protein inhibitor (FAPI) positron emission tomography (PET) for oncologic and fibroinflammatory diseases. FAP is expressed broadly on activated mesenchymal cells—including cancer-associated fibroblasts (CAFs) and myofibroblasts within desmoplastic tumor stroma, FAP-positive tumor cells in selected sarcomas, and activated fibroblasts in chronic fibroinflammatory disorders such as rheumatoid arthritis, Crohn’s disease, and organ fibrosis. By targeting these activated fibroblasts, [^68^Ga]- and [^18^F]-labeled FAPI tracers provide high tumor-to-background contrast, particularly in desmoplastic and stromal-rich cancers. Compared with [^18^F]FDG, FAPI PET demonstrates superior lesion conspicuity in selected malignancies and enables a streamlined, non-fasting imaging workflow. Beyond oncology, FAPI PET is emerging as a promising tool for assessing cardiac fibrosis, pulmonary inflammation, and autoimmune conditions characterized by fibroblast activation. A systematic literature search of PubMed and Scopus was performed for peer-reviewed publications from 1 January 2018 to 28 February 2026. Inclusion criteria encompassed original studies, systematic reviews, meta-analyses, clinical guidelines, case series, and case reports reporting on FAPI-targeted PET in human subjects or translational models, published in English. After screening, 256 sources met the eligibility criteria and are included. The development of standardized SNMMI/EANM imaging protocols, along with ongoing multicenter trials and the first prospective phase 2 clinical trial of ^68^Ga-FAPI-46 PET with histopathological confirmation, now supports the reproducible implementation of FAPI PET across institutions. FAPI PET demonstrates strong translational potential, largely due to its favorable biodistribution, safety profile, and theranostic flexibility. However, its widespread use in routine clinical practice is contingent upon large-scale clinical validation, structured reader training, and formal regulatory approval. In conclusion, FAPI PET represents a maturing molecular imaging platform targeting activated fibroblasts across oncologic and fibroinflammatory diseases. Its widespread adoption into clinical practice requires large-scale prospective trials, reader training, standardized reporting, and regulatory approval—all of which are now actively underway.

## 1. Introduction

Fibroblast activation protein (FAP) has recently emerged as a promising molecular imaging target, leading to the development of FAP inhibitor (FAPI) tracers. These agents provide distinct advantages over conventional [^18^F]fluorodeoxyglucose ([^18^F]FDG) imaging by visualizing stromal rather than metabolic tumor activity. With rapidly expanding clinical evidence, indications extending beyond oncology, and the publication of Society of Nuclear Medicine and Molecular Imaging (SNMMI) and European Association of Nuclear Medicine (EANM) procedure standards, an updated synthesis of this rapidly evolving field is therefore timely.

This scoping review summarizes the biological rationale, tracer development, oncologic and non-oncologic applications, theranostic potential, and future perspectives of FAPI PET, emphasizing its evolving role in precision medicine. The review was conducted and reported in accordance with PRISMA-ScR (Preferred Reporting Items for Systematic Reviews and Meta-Analyses Extension for Scoping Reviews) guidelines [see Methods].

FDG PET has long served as the cornerstone of oncologic molecular imaging, exploiting the Warburg effect to visualize tumor glucose metabolism [1,2]. However, FDG lacks specificity and performs poorly in certain tumor types, such as pancreatic [3]. Uptake in inflammation, infection, and post-therapeutic changes frequently yields false positives, prompting the search for alternative molecular targets such as the tumor stroma [3,4,5]. These limitations have driven the development of next-generation radiopharmaceuticals that interrogate alternative biological pathways beyond glucose metabolism, aiming for improved specificity and diagnostic reliability [6].

### 1.1. The Tumor Microenvironment (TME) as a Target

The tumor microenvironment (TME)—a complex ecosystem of cells and matrix components—critically influences tumor progression, invasion, and metastasis [7,8,9,10]. Within this niche, FAP—a type II transmembrane serine protease expressed by cancer-associated fibroblasts (CAFs)—has gained attention as a highly specific imaging and therapeutic target.

Originally identified as the F19 antigen, FAP is expressed on reactive stromal fibroblasts in epithelial cancers but is largely absent from normal adult tissues, underscoring its tumor specificity [11,12,13,14]. Beyond structural support, CAFs actively modulate the tumor immune milieu, facilitate immune evasion, and promote therapeutic resistance [8,15,16]. Their activity is intimately linked to epithelial–mesenchymal transition (EMT) processes, in which FAP-expressing fibroblasts drive tumor invasion and resilience against therapy [17]. By imaging this stromal component, FAPI PET enables visualization of tumor biology that cannot be captured by metabolism-based tracers like [^18^F]FDG.

### 1.2. Fibroblast Activation Protein (FAP) Biology

Fibroblast activation protein (FAP) is a type II transmembrane serine protease belonging to the dipeptidyl peptidase IV (DPP4/CD26) enzyme family and exhibits both dipeptidyl peptidase and endopeptidase activity [18]. Through cleavage of proline-containing peptide bonds, FAP contributes to extracellular matrix degradation and facilitates tumor invasion [14]. FAP upregulation reflects tissue remodeling pathways driven by TGF-β signaling and extracellular matrix turnover. Early pharmacologic studies with small-molecule inhibitors such as PT-100 demonstrated antitumor and immunomodulatory effects, establishing a foundation for FAP-targeted therapeutics [19]. FAP expression is largely restricted to activated fibroblasts within the stroma of epithelial malignancies and in tissues undergoing remodeling (e.g., wound healing, fibrosis), while its presence in normal adult tissue remains minimal [20,21,22]. Beyond cancer-associated fibroblasts, FAP is also expressed by activated mesenchymal cell populations, including FAP-positive tumor cells in selected sarcoma subtypes, activated synoviocytes in rheumatoid arthritis, intestinal fibroblasts in Crohn’s disease strictures, and myofibroblasts in organ fibrosis. This generalized pathological fibroblast activation—rather than strictly tumor-specific expression—is what defines FAPI PET’s broad clinical scope across oncologic and non-oncologic disease [23].

## 2. Methods

This scoping review was designed, conducted, and reported in accordance with the PRISMA Extension for Scoping Reviews (PRISMA-ScR) guidelines. The PRISMA-ScR checklist is provided as Table 1.

**Table 1:** PRISMA-ScR Checklist Summary.

PRISMA-ScR Item	Description	Location in Manuscript	Status
Title	Indicate that the document is a scoping review.	Title states: “A Narrative Scoping Review”	Item 1—Met
Abstract	Provide a structured summary including objective, eligibility criteria, sources of evidence, charting methods, results, and conclusions.	Structured abstract present; PRISMA-ScR compliance stated.	Item 2—Met
Rationale	Describe the rationale for the review in the context of what is already known.	Provided in Introduction.	Item 3—Met
Objective	Provide an explicit statement of the questions being addressed.	Stated in Introduction.	Item 4—Met
Protocol and registration	Indicate whether a review protocol exists; provide registration number if registered.	Not pre-registered. Protocol available from corresponding author on request.	Item 5—Reported
Eligibility criteria	Specify characteristics of included sources and rationale.	Described in Methods (Section 2.2).	Item 6—Met
Information sources	Describe all information sources in the search.	PubMed and Scopus; hand-searching; guidelines.	Item 7—Met
Search	Present full electronic search strategy for ≥1 database.	Described in Methods (Section 2.3).	Item 8—Met
Selection of sources	State process for selecting sources of evidence.	Described in Methods (Section 2.4).	Item 9—Met
Data charting	Describe data charting methods.	Described in Methods (Section 2.5).	Item 10—Met
Data items	List and define all variables for which data were sought.	Described in Methods (Section 2.5).	Item 11—Met
Results of individual sources	Provide a summary table of included sources.	Table 2, Table 3 and Table 4 provide evidence summaries.	Item 12—Met
Synthesis of results	Describe the methods of handling and summarizing data.	Described in Methods (Section 2.6); narrative synthesis used.	Item 13—Met
PRISMA-ScR flow diagram	Provide a flow diagram of screening process.	Figure 1 (PRISMA-ScR Flow Diagram).	Item 14—Met
Limitations	Discuss limitations of the scoping review process.	Described in Limitations section.	Item 15—Met
Conclusions	Provide a general interpretation and implications for future research.	Described in Conclusion.	Item 16—Met
Funding	Describe sources of funding.	No external funding.	Item 17—Met

**Figure 1:**
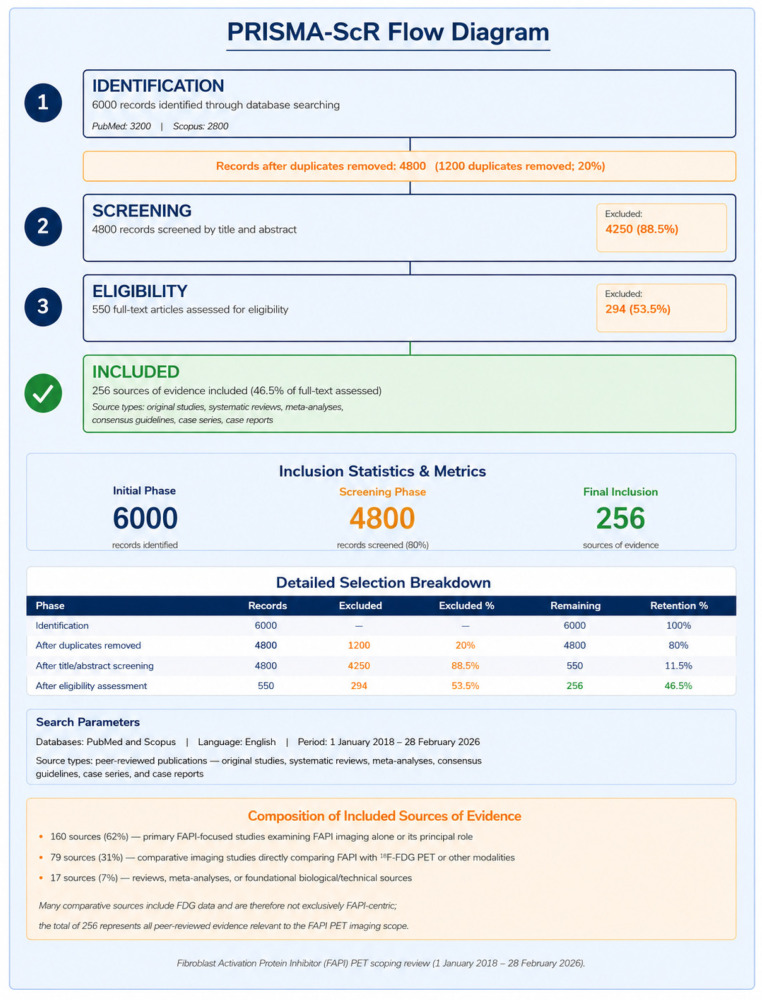
PRISMA-ScR Flow Diagram for this scoping review.

### 2.1. Protocol and Registration

A formal review protocol was not pre-registered with a public registry prior to commencing data extraction. This represents a limitation acknowledged in accordance with PRISMA-ScR item 5. The protocol and data charting framework are available from the corresponding author upon reasonable request. Future updates of this review will be prospectively registered with PROSPERO or OSF prior to data extraction, in alignment with MDPI and PRISMA-ScR recommendations.

### 2.2. Eligibility Criteria

Sources of evidence were eligible for inclusion if they: (1) reported on FAPI-targeted PET imaging in human subjects or well-validated preclinical models with direct translational relevance; (2) addressed oncologic or non-oncologic clinical applications, tracer development, standardization, or theranostics; (3) were published in English between January 2018 and February 2026; and (4) were peer-reviewed original studies, systematic reviews, meta-analyses, consensus guidelines, case series, or case reports. Editorials without primary data, non-English publications, conference abstracts without a companion full publication, and studies exclusively addressing SPECT-based FAP imaging without PET comparators were excluded. Dosimetry studies reporting radiation burden or pharmacokinetics of FAPI tracers were included when they provided clinical translation data.

### 2.3. Information Sources and Search Strategy

A systematic literature search was conducted in PubMed and Scopus from 1 January 2018 to 28 February 2026. The year 2018 was chosen as the lower boundary, corresponding to the first in-human FAPI PET publications. The following search string was applied (with appropriate MeSH terms and field tags for PubMed, and equivalent strategies for Scopus):

*(“fibroblast activation protein” OR “FAP inhibitor” OR “FAPI” OR “FAP-targeted”) AND (“PET” OR “PET/CT” OR “PET/MRI” OR “positron emission tomography”)*

Additional filters applied: human studies (with animal studies included only when directly informing clinical translation), English language, and full-text availability. Hand-searching of reference lists of identified systematic reviews and key review articles was performed to identify additional sources. The SNMMI/EANM procedure standard and society guidelines were searched directly from their respective society websites. A supplementary search using the terms “FAP-RADS,” “FAPI theranostics,” and “FAPI radioligand therapy” was performed to ensure comprehensive coverage of subspecialty topics.

### 2.4. Selection of Sources of Evidence

Titles and abstracts of identified records were screened by one reviewer (E.P.) against the eligibility criteria. Full texts of potentially eligible sources were then retrieved and assessed. A PRISMA-ScR flow diagram illustrating the number of records identified, screened, assessed for eligibility, and included is provided as Figure 1. Disagreements in eligibility were resolved through consensus discussion. Given the narrative synthesis design and the large heterogeneity of study types and populations, a formal inter-rater reliability assessment was not performed; instead, a single experienced reviewer with expertise in nuclear medicine and molecular imaging undertook primary screening, with all included sources independently verified against inclusion criteria.

### 2.5. Data Charting

A standardized data charting form was used to extract the following variables from included sources: (1) author(s) and year; (2) study design (prospective/retrospective, phase, sample size); (3) FAPI tracer(s) evaluated; (4) comparator (typically FDG PET); (5) clinical indication/tumor type; (6) key outcome measures (sensitivity, specificity, PPV, SUVmax, tumor-to-background ratio, staging impact, therapeutic management changes); and (7) main conclusions and limitations. For non-oncologic studies, additional variables were captured, including the specific fibrotic or inflammatory condition, imaging biomarker used, and correlation with clinical or histopathological outcomes.

### 2.6. Synthesis of Results

Given the marked heterogeneity in study design, patient populations, FAPI tracers used, and outcome metrics, a formal quantitative meta-analysis was not performed. Instead, a narrative synthesis was conducted, grouping findings thematically by clinical application domain: (1) tracer development and pharmacokinetics; (2) standardization and guidelines; (3) oncologic applications (by tumor type); (4) non-oncologic applications; (5) theranostics; (6) imaging pitfalls; (7) digital/LAFOV technology; and (8) AI and radiomics. Table 2, Table 3 and Table 4 provide structured evidence summaries for key clinical domains. All numerical data cited are drawn directly from original publications as referenced. Note: references are numbered in order of first citation in the manuscript text; table references carry numbers matching their first in-text citation.

## 3. Results

### 3.1. PRISMA-ScR Flow Diagram

The systematic database search identified 3200 records in PubMed and 2800 records in Scopus (total 6000 records). After duplicate removal, 4800 records were screened by title and abstract. Following exclusion of records not meeting eligibility criteria (4250 records, 88.5%), 550 full-text articles were assessed for eligibility. After full-text review, 256 sources of evidence were included in this scoping review (46.5% of full-text assessed). Of these, 160 (62%) were primary FAPI-focused studies, 79 (31%) were comparative imaging studies (FAPI versus [^18^F]FDG PET or other modalities), and 17 (7%) were reviews, meta-analyses, or foundational biological or technical sources. The PRISMA-ScR flow diagram is presented in Figure 1 above.

### 3.2. Development of FAP-Targeted Imaging Agents

Reactive tumor stroma may constitute up to 90% of total tumor volume in cancers such as pancreatic ductal adenocarcinoma, making stroma-oriented molecular imaging a potentially superior strategy compared with targeting malignant cells directly [24,25,26]. This concept has driven the design of radiolabeled FAP inhibitors and stimulated extensive investigation into FAP-specific imaging for oncology and fibroinflammatory disorders [19,27,28,29].

FAP’s central role in extracellular matrix remodeling and immune modulation has positioned it as an important diagnostic and therapeutic target [15,23]. Early therapeutic approaches using monoclonal antibodies confirmed the safety of FAP targeting but suffered from limited tumor penetration and suboptimal pharmacokinetics, leading to a transition toward small-molecule inhibitors with enhanced bioavailability and tissue retention.

The modern generation of FAP-targeted PET tracers began with quinoline-based inhibitors, which demonstrated high affinity for FAP and favorable biodistribution [30,31,32,33]. Subsequent optimization produced next-generation FAPI compounds incorporating squaramide-coupled bifunctional chelators that improved metabolic stability and tumor residence time [34,35,36]. These modifications also enable labeling with multiple isotopes, broadening both diagnostic and therapeutic applications [37].

Table 2 summarizes the principal FAPI tracers currently in clinical and preclinical use, illustrating progressive optimization of pharmacokinetics and radionuclide versatility.

Recent innovations include a ^68^Ga/^177^Lu-labeled theranostic pair with improved tumor uptake and retention, a trifunctional FAPα ligand with dual imaging and therapeutic capability and dimeric constructs demonstrating superior tumor residence time [38,39,40]. Additionally, to overcome rapid clearance, albumin-binder conjugates such as TEFAPI-06 and TEFAPI-07 have been developed, showing significantly enhanced tumor retention and promising therapeutic efficacy [41]. Collectively, these advances have established FAPI PET as a robust molecular imaging platform targeting tumor stroma rather than tumor metabolism (Figure 1) [32,33]. Comprehensive reviews have summarized the evolution, clinical applications, and remaining challenges of this rapidly expanding field [42].

Recent developments in ^18^F-based FAPI chemistry have advanced clinical accessibility. Zhang et al. developed a novel [^18^F]AlF-H_3_RESCA-FAPI radiotracer utilizing aluminum fluoride (AlF) chelation at room temperature, achieving high radiochemical yield and FAP-specific binding comparable to ^68^Ga-FAPI tracers. Preclinical evaluation demonstrated rapid tumor accumulation with excellent tumor-to-background ratios in U87MG xenografts. The ^18^F-labeling approach offers extended shelf-life (110 min vs. ^68^Ga 68 min) and simplified kit-based synthesis, enhancing clinical accessibility [43].

**Table 2:** Summary of Principal FAPI Tracers.

Tracer	Chemical Scaffold/Type	Radionuclide(s)	Key Properties	Representative Study
FAPI-02	Quinoline-based gly-cyanopyrrolidine small-molecule inhibitor	^68^Ga	First-generation compound; high FAP affinity, moderate tumor retention; proof-of-concept for in-human studies	Loktev et al. (2018) [32]
FAPI-04	Quinoline-based derivative with optimized linker	^68^Ga/^18^F	Rapid blood clearance, high tumor-to-background contrast, reproducible biodistribution; widely validated clinically	Giesel et al. (2019) [30]
FAPI-46	Quinoline-based, modified spacer	^68^Ga	Improved tumor retention and contrast; preferred diagnostic tracer; phase 2 clinical trial validated accuracy with histopathology [44]	Lindner et al. (2018) [33]
FAPI-74	Quinoline-based, Al-^18^F chelation	^18^F	Longer half-life permits centralized production and distribution; equivalent diagnostic accuracy to ^68^Ga analogues; phase 3 trial ongoing	Novruzov et al. (2023) [45]
FAPI-42	Quinoline-derived	^68^Ga	Slightly prolonged tumor residence compared with FAPI-46; under evaluation for therapy planning	Mu et al. (2023) [46]
FAP-2286	Peptide-based DOTA conjugate	^68^Ga/^177^Lu/^90^Y	High affinity and prolonged retention; under clinical investigation for theranostic use	Baum et al. (2022) [47]
Albumin-binder conjugates (TEFAPI series)	Quinoline core with albumin-binding motif	^68^Ga/^177^Lu	Increased circulation time and tumor residence; promising for therapeutic radionuclides	Xu et al. (2022) [41]
LNC1007	Dual-targeting FAPI-RGD heterodimer	^68^Ga	Alternative scaffold with favorable dosimetry and comparable affinity	Zang et al. (2023) [48]
[^18^F]AlF-H_3_RESCA-FAPI	H_3_RESCA-chelator conjugated to FAPI	^18^F	Room-temperature synthesis; extended shelf-life (110 min); high radiochemical yield; FAP-specific binding	Zhang et al. (2025) [43]

### 3.3. Clinical Translation of FAPI PET

The clinical implementation of FAPI PET has been accelerated by the joint SNMMI/EANM procedure standards, which harmonize patient preparation, administered activity, acquisition parameters, and interpretation criteria [49]. These guidelines promote standardized imaging across centers and emphasize correlation with morphological studies to avoid pitfalls related to physiological or inflammatory uptake.

FAPI PET has evolved from a novel tracer concept into a rapidly maturing clinical tool supported by reproducible protocols and expanding indications. Its high tumor-to-background ratios result from selective accumulation in FAP-expressing stroma and rapid clearance from blood and non-target tissues [21,30,50]. This profile enhances lesion conspicuity, particularly in cancers where stromal biology dominates over glucose metabolism.

**Figure 2:**
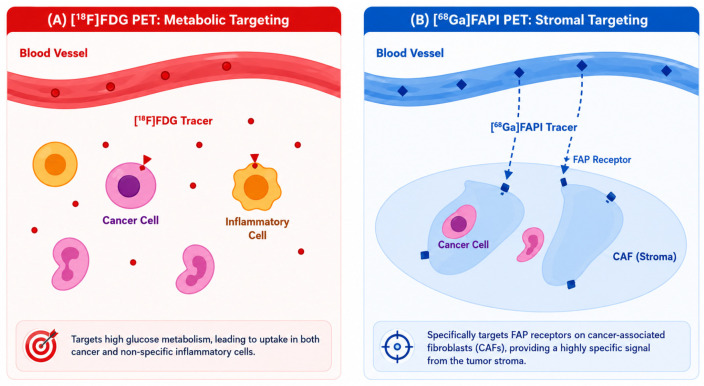
Conceptual Comparison of [^18^F]FDG and [^68^Ga]FAPI PET Imaging Mechanisms. (**A**) FDG PET targets cellular glucose metabolism. Uptake is high in malignant cells but also in non-malignant inflammatory cells (e.g., macrophages) and tissues with high metabolic rates, leading to potential false positives. (**B**) FAPI PET targets Fibroblast Activation Protein (FAP) expressed on cancer-associated fibroblasts (CAFs) within the tumor microenvironment. This stromal targeting provides high tumor-to-background contrast and greater specificity, as FAP expression is low in most normal tissues and non-specific inflammatory sites. Created by the authors. No copyright permission required.

Biodistribution and dosimetry studies consistently show low background activity and excellent image contrast in pancreatic, gastric, head-and-neck, and colorectal cancers [51,52,53,54,55]. Comparative analyses demonstrate higher lesion detection than [^18^F]FDG, notably in pancreatic ductal adenocarcinoma and peritoneal metastases, with meta-analyses confirming improved sensitivity and staging accuracy [56]. Early reports in lymphoma suggest a complementary role across subtypes with variable FAP expression [57]. Beyond oncology, FAPI PET enables non-invasive assessment of fibroblast activation in fibrotic and inflammatory disorders, including pulmonary fibrosis, inflammatory bowel disease, and post-infarct cardiac remodeling (Figure 2) [58,59,60,61]. Simplified workflow, absence of dietary preparation, and a favorable safety profile support clinical translation. Initial experiences with FAP-targeted radioligand therapy (RLT) have shown encouraging safety and preliminary efficacy [47,62]. A recent systematic review confirmed high diagnostic accuracy and broad translational promise [63].

### 3.4. Clinical Guidelines and Standardization Frameworks

The publication of the joint SNMMI/EANM procedure standard for FAPI imaging represents a major step toward harmonized implementation [49]. This consensus document defines evidence-based recommendations for patient preparation, radiopharmaceutical administration, image acquisition, and reporting, thereby ensuring reproducibility across institutions and supporting both clinical use and research trials.

Key procedural aspects include standardized administered activities of approximately 100–200 MBq for ^68^Ga-labeled compounds and corresponding effective doses of 0.010–0.015 mSv/MBq. FAPI imaging does not require fasting or glucose monitoring, which simplifies workflow relative to [^18^F]FDG. Physiologic biodistribution—most prominent in kidneys, urinary bladder, and uterus—must be recognized to avoid false-positive interpretation. The guidelines emphasize correlation with morphological imaging (CT or MRI) and clinical context to differentiate benign fibrotic or inflammatory uptake from malignancy [64]. Quality control (QC) and quality assurance (QA) procedures are integral to maintaining quantitative accuracy. Medical physicists ensure scanner calibration, radiation-dose optimization, and compliance with international performance standards. Physicians supervising or interpreting FAPI PET should be board-certified in nuclear medicine and familiar with both the biological behavior of FAP-expressing lesions and potential pitfalls due to non-malignant uptake [65,66].

## 4. Expanded Clinical Applications

**Figure 3:**
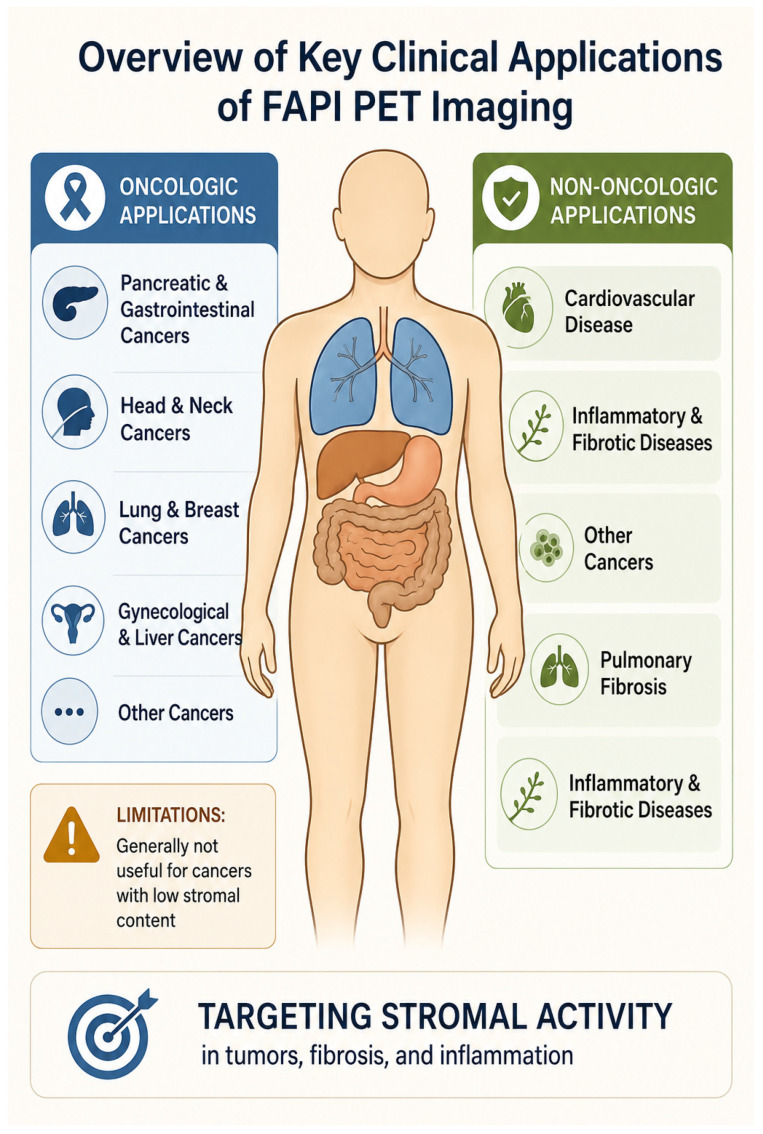
Overview of Key Clinical Applications of FAPI PET Imaging. This figure highlights the broad utility of FAPI PET in both oncologic and non-oncologic diseases, targeting fibroblast activation in tumors, fibrosis, and inflammation. Created by the authors. No copyright permission required.

The expanding clinical adoption of FAPI PET spans a wide spectrum of oncologic and fibroinflammatory diseases. Initially conceived for oncology, its favorable biodistribution, rapid blood clearance, and high target-to-background ratios have facilitated broader exploration in malignancy, organ fibrosis, and inflammatory remodeling. Figure 3 provides an overview of the principal oncologic and non-oncologic clinical applications of FAPI PET, highlighting its utility in stromal-rich malignancies, cardiovascular disease, and fibroinflammatory disorders.

### 4.1. Oncologic Applications

FAPI PET’s oncologic applications depend primarily on the tumor’s stromal activity, FAP expression and the desmoplastic reaction [14,24,67]. This makes FAPI PET particularly effective for stromal-rich malignancies [68]. Table 3 provides an executive summary of selected recent advancements by tumor type.

**Table 3:** Executive Summary of Selected Recent Advancements in FAPI PET Imaging.

Tumor Type	FDG PET—Limitations/ Strengths	FAPI PET—Strengths/ Advantages	Clinical Implication	References
Pancreatic adenocarcinoma	Often limited by high background uptake in bowel; variable FDG avidity	Higher tumor-to-background ratio; better peritoneal metastasis detection	Improved detection of small/peritoneal lesions and impact on management	Giesel et al. (2019) [30]; Kratochwil et al. (2019) [51]; Chen et al. (2020) [52]; Pang et al. (2021) [53]
Cholangiocarcinoma	Low uptake in many cases, leading to diagnostic challenges	High FAPI uptake due to dense desmoplastic stroma, providing clear visualization	Superior staging; better detection of small intrahepatic and nodal metastases	Pabst et al. (2023) [69]; Shi et al. (2021) [70]
Breast cancer (esp. lobular type)	Heterogeneous FDG uptake, with low affinity in subtypes like invasive lobular carcinoma	Consistently high uptake across subtypes; strong detection of bone lesions	Enhanced sensitivity in low-FDG-avid subtypes; improved skeletal staging; PET/MRI complementarity	Guo et al. 2025 [71] Kömek et al. 2021 [72]
Sarcoma	Variable FDG uptake by subtype	Strong FAPI uptake in some high-grade and fibrotic tumors (CAF-rich)	More accurate disease burden assessment in selected subtypes; potential selection for FAP-targeted therapy	Lindner et al. (2018) [33]; Kratochwil et al. (2019) [51]
Colorectal cancer (peritoneal metastases)	FDG limited by physiologic bowel uptake; low sensitivity for small lesions	High sensitivity for detecting small peritoneal implants	Improved surgical planning, more accurate staging, and treatment response assessment	Chen et al. (2020) [52], Pang et al. (2021) [53];
Gastric cancer	FDG often negative in diffuse/mucinous types; physiologic uptake obscures lesions	High FAPI uptake in stromal-rich tumors, including signet-ring cell carcinoma	Superior primary tumor detection and nodal staging; prognostic implications of uptake intensity	Chen et al. (2023) [73]; Beyhan et al. (2024) [74]; Gündoğan et al. (2022) [75]; Zhang et al. (2022) [76]
Head and Neck cancers	Confounded by physiologic FDG uptake in muscles, glands, and inflammation	Higher lesion-to-background contrast; superior mapping of nodal disease	Better staging, improved detection of unknown primary tumors, radiotherapy planning	Syed et al. (2020) [77]; Kratochwil et al. (2019) [51]
Glioblastoma	High physiologic glucose uptake in brain limits FDG	Low background in normal brain tissue, high tumor contrast	Useful for evaluating both primary and recurrent gliomas	Röhrich et al. (2020) [78]
Liver metastases (various origins)	FDG may miss small or necrotic lesions; high liver background	Low FAPI uptake in healthy parenchyma yields excellent contrast	Superior detection of both primary and metastatic hepatic lesions, including post-treatment	Giesel et al. (2019) [30]; Chen et al. (2020) [52]

#### 4.1.1. Stromal-Rich Malignancies

These tumors are characterized by high concentrations of CAFs and a dense stromal matrix, making them ideal targets for FAPI imaging. This category includes a wide range of common cancers.

- Pancreatic Cancer: In pancreatic ductal adenocarcinoma (PDAC), the desmoplastic stroma can comprise up to 90% of the tumor volume [15,79]. This stromal matrix prominently features FAP-expressing CAFs, which are known to influence fibrosis, tumor spread, and resistance to therapy [15]. This biological underpinning has been further validated by studies directly correlating in vivo FAPI uptake with ex vivo immunohistochemical FAP expression in both PDAC and its precursor lesions [79]. Several studies have shown that FAPI can differentiate PDAC from pancreatitis with high sensitivity and specificity [80,81,82,83]. It is also valuable for clarifying equivocal FDG findings and can impact radiotherapy planning [55,84,85]. In a prospective, intra-individual comparison in patients with pancreatic ductal adenocarcinoma (PDAC), FAPI-74 imaging detected up to 22% more lesions than FDG and successfully identified a primary tumor that was missed [45].
- Gastrointestinal Cancers: For gastric and colorectal cancers, FAPI has demonstrated heightened sensitivity for detecting primary tumors and, most notably, peritoneal metastases, which are often occult on other imaging modalities [62,86,87,88]. A meta-analysis by Huang et al. confirmed superior detection sensitivity of FAPI over FDG in several gastrointestinal malignancies [89]. A systematic review and meta-analysis by Liu et al. confirmed the utility of FAPI for diagnosing both primary and metastatic lesions in abdominal and pelvic malignancies [90]. Comparative studies focusing on broad gastrointestinal carcinomas have found FAPI useful for detecting both primary and metastatic disease, with one study concluding that it provides better diagnostic imaging than FDG for malignant colorectal carcinomas [91,92,93,94]. Its value is especially pronounced in FDG-negative subtypes such as gastric signet-ring cell carcinoma [73]. Further highlighting this, Wang et al. demonstrated a specific imaging strategy using FAPI-04 PET/MR for managing Krukenberg tumors originating from gastric signet-ring-cell carcinoma [95]. Furthermore, beyond improving detection and staging, initial findings suggest that the intensity of FAPI uptake carries prognostic value in patients with gastric cancer [74,75,76]. A recent meta-analysis confirmed that FAPI demonstrates a higher sensitivity (0.82) versus FDG (0.51) for diagnosing lymph node metastasis in digestive system cancers [96]. A separate meta-analysis confirmed its superiority over FDG for detecting peritoneal metastases, and another study affirmed its value for malignant colorectal carcinomas [90,97]. In gastrointestinal and gynecological malignancies, FAPI has demonstrated superior detection of peritoneal metastases compared with FDG, an advantage confirmed in recent head-to-head meta-analyses [56]. Head-to-head comparative studies have demonstrated that FAPI outperforms FDG in detecting peritoneal and nodal metastases in colorectal cancer, notably enhancing staging accuracy [98]. This imaging advantage is rooted in tumor biology, as pathological studies in colorectal cancer confirm that FAP expression is significantly increased at the tumor’s invasive front and is associated with a higher tumor-stroma ratio [99].
- Head and Neck Cancers: By targeting the substantial stromal component common in head and neck squamous cell carcinoma (HNSCC), FAPI provides a distinct diagnostic advantage over conventional imaging. It yields higher-contrast visualization and reduces the false-positive findings associated with FDG for regional nodal metastases. Studies have shown it can successfully detect primary tumors and nodal metastases that FDG may miss, especially in oral squamous cell carcinoma [51,100,101]. For other subsites, a 2025 head-to-head comparison evaluated FAPI-42 against FDG specifically in laryngeal squamous cell carcinoma [102]. In nasopharyngeal carcinoma, FAPI more clearly visualizes disease, particularly small-volume metastases, due to lower physiological background uptake in the nasopharynx compared to FDG [103]. Furthermore, it has shown significant promise in identifying the primary tumor in cases of head and neck cancer of unknown primary, outperforming conventional imaging methods [101]. It also aids in radiotherapy planning for Waldeyer’s tonsillar ring malignancies and adenoid cystic carcinomas by providing superior disease mapping [104,105].
- Lung Cancer: While FDG is the standard for non-small cell lung cancer (NSCLC) staging, its specificity suffers in regions with co-existing inflammatory lung disease [3]. FAPI, with its characteristically low uptake in inflamed tissue, provides increased sensitivity for primary tumors and more accurate characterisation of mediastinal and hilar lymph nodes and pleural involvement [106,107,108,109]. Studies have specifically validated the accuracy of FAPI for lymph node metastasis in NSCLC and have demonstrated a direct correlation between tracer uptake and the histological expression of FAP in the cancerous tissue [110]. A recent meta-analysis supports its high diagnostic performance for lymph node metastases [111]. In malignant pleural mesothelioma, FAPI consistently uncovers regions of spread that are too subtle for FDG scans [112]. Furthermore, pretreatment FAPI uptake is emerging as a potential biomarker for predicting immunotherapy outcomes in NSCLC [113,114]. Two recent reviews provided a thorough summary of the performance and future prospects of FAPI scans in lung cancer [115,116].
- Breast Cancer: Radiolabeled FAPI may improve the detection, staging, and assessment of treatment response in breast cancer [71,117]. FAPI has shown increased FAP ligand uptake independent of the histological phenotype or molecular subtype [71]. It has proven particularly effective in subtypes where FDG struggles, such as invasive lobular carcinoma and other breast cancers with low FDG affinity [118,119]. Multiple studies have shown that FAPI detects both primary and metastatic lesions, especially bone metastases, with greater sensitivity than FDG in patients with low-FDG-avid tumors [72,119,120,121,122]. A systematic review focused specifically on bone metastases confirmed the superior diagnostic performance of FAPI compared to FDG across a range of different cancers, reinforcing its value for skeletal staging [123]. The use of simultaneous FAPI PET/MRI protocols has demonstrated complementary high-contrast molecular and anatomical data, particularly valuable for mapping multifocal breast cancer [124].
- Gynecological Malignancies: For ovarian and uterine cancers, peritoneal carcinomatosis is a principal determinant of outcome [125,126]. Radiolabeled FAPI may improve the detection, staging, and assessment of treatment response in the most common gynecological malignancies [117,121]. FAPI has demonstrated remarkable proficiency in detecting small peritoneal implants that are often invisible on FDG due to physiological bowel activity [127,128,129]. Prospective studies show detection rates for peritoneal implants exceeding 95% [127]. It also offers a nuanced assessment of lymph node involvement in cervical and endometrial cancers [127,130]. In recurrent disease, it can help differentiate active tumor from post-treatment fibrosis [130].
- Liver and Hepatobiliary Cancers: Primary liver tumors like hepatocellular carcinoma (HCC) and intrahepatic cholangiocarcinoma (ICC) pose diagnostic challenges, with practice guidance available for standard management [131,132]. FAPI capitalises on the typically low background uptake in healthy liver parenchyma, allowing for clear visualisation of these tumors, even in cases with low glucose metabolism [133,134,135,136,137,138]. In patients with cholangiocarcinoma, a study by Pabst et al. found that FAPI-46 offered superior tumor detection compared to both FDG and conventional CT [69]. A clinical prospective study by Jinghua et al. specifically investigated FAP inhibitor for the diagnosis of biliary tract carcinoma, providing important new data for this application [139]. Comparative studies demonstrate substantial gains in sensitivity over FDG, particularly for well-differentiated or FDG-non-avid lesions, and show prognostic value for HCC [134,135,140,141]. Similarly, a 2021 prospective pilot study from Shi et al. compared FAPI with FDG for the diagnosis of primary hepatic tumors [70]. It is also valuable for identifying viable tumor tissue after local regional treatment [142]. A recent literature review evaluated the diagnostic performance of FAPI in patients with both primary and metastatic liver tumors [143]. Prospective evaluation of FAPI in hepatobiliary tumors has further validated its superior sensitivity for challenging lesions, including small intrahepatic and nodal metastases [144]. Further supporting this, a head-to-head comparison using the dual-targeting FAPI-RGD tracer 68Ga-LNC1007 also found it was superior to 18F-FDG for diagnosing hepatocellular carcinomas [48].
- Thyroid Cancer: The investigation of FAPI in thyroid cancer is supported by biological evidence showing that cancer-associated fibroblasts are positively correlated with tumor dedifferentiation and aggressiveness [145]. For patients with radioiodine-refractory differentiated thyroid cancer, FAPI addresses a critical diagnostic challenge: localising recurrent disease when traditional iodine scans are negative despite rising serum thyroglobulin levels [146,147]. It shows high sensitivity for detecting elusive bone and lymph node metastases [148,149,150]. Focusing specifically on papillary thyroid cancer, Han et al. evaluated the diagnostic value of FAPI-04 PET/MRI for assessing lymph node metastasis [151]. In head-to-head comparisons, FAPI routinely identifies additional lesions not visualised by FDG [46,152]. Preliminary data also suggest a role in staging medullary thyroid cancer, with recent trials demonstrating its potential for accurate management [153,154,155,156]. A 2024 systematic review assesses FAPi-based agents in thyroid cancer, framing them as a new step towards improved diagnosis and therapy [157].
- Urothelial Cancer: In a head-to-head intra-individual comparison, FAPI demonstrated significantly higher lesion uptake and improved lesion detection rates compared to FDG, particularly in nodal and osseous disease sites [158]. Initial clinical experience with the novel tracer FAP-2286 in patients with muscle-invasive and metastatic urothelial carcinoma showed high sensitivity for detecting small-volume disease [159].
- Other Cancers: Peritoneal imaging represents a key application area where bowel activity limits FDG utility, with demonstrated benefits in ovarian and gastric cancer assessment [129,160,161,162]. FAPI uptake is observed in metastatic brain tumors and gliomas, though the benefit over standard MRI for primary brain tumors remains less clear. Studies confirm that the expression and enzymatic activity of FAP in human astrocytic tumors are associated with tumor grade [163,164,165,166,167]. Initial studies in gliomas show elevated uptake corresponding to high-grade histology [78,168]. A pilot study assessed the performance of FAPI in evaluating glioblastoma specifically before radiotherapy [169]. The application of FAPI is also being explored in rarer tumors, such as adrenocortical carcinoma [170].

#### 4.1.2. Cancer of Unknown Primary (CUP)

Cancer of unknown primary (CUP) accounts for approximately 3–5% of all malignant epithelial tumors and is associated with a median survival of less than 12 months [171]. Conventional staging frequently fails to localize the primary lesion, limiting access to site-directed therapy. Historical FDG baseline studies established the limitations of conventional PET imaging in detecting occult primary tumors in CUP. A seminal review by Rusthoven et al. analyzing 16 studies (302 patients) demonstrated that FDG-PET achieved variable detection rates in identifying primary tumors in cervical metastases from unknown origin. These findings establish FDG-PET as a baseline comparator for novel imaging modalities such as FAPI PET/CT [172]. Because FAPI PET targets the activated stroma rather than tumor metabolism, it offers a histology-agnostic mechanism for primary tumor identification regardless of cellular origin.

In the prospective HNCUP study by Gu et al., ^68^Ga-FAPI PET demonstrated superior performance to FDG PET for localizing the primary tumor in head-and-neck CUP, with sensitivity of 51% versus 25%, positive predictive value of 98% versus 43%, and treatment-altering management changes in 24% of patients [101]. These findings support FAPI PET as a problem-solver in CUP and have prompted an ongoing prospective trial (NCT05263700).

#### 4.1.3. Rare and Uncommon Tumor Entities

Beyond the principal tumor categories, FAPI PET has been applied to rare malignancies in which conventional imaging is often inconclusive. In the first dedicated rare-cancer FAPI PET cohort, Dendl et al. evaluated 55 patients across multiple uncommon entities, including cancers of unknown primary, rare head-and-neck malignancies, biliary–pancreatic tumors, urinary-tract cancers, and neuroendocrine tumors, demonstrating high tracer uptake in primary lesions (mean SUVmax 10.1) and metastases—particularly peritoneal carcinomatosis (mean SUVmax 9.8, tumor-to-background ratio 29.6)—and supporting FAPI PET as a useful tool for staging rare malignancies that are often missed by FDG PET [173]. These rare entities are also represented in Table 3.

#### 4.1.4. Tumors with Low/Variable FAP Expression

Several cancer types do not consistently induce strong FAP uptake, including most lymphomas, multiple myeloma, prostate adenocarcinoma, renal cell carcinoma, melanoma, and seminoma [50,51]. FAPI is therefore unlikely to play a significant role in staging most of these cancers [51,174]. However, early studies in multiple myeloma report enhanced visualisation of osseous involvement compared to FDG [154,175].

#### 4.1.5. Tumors of Mesenchymal Origin

Sarcomas are of particular interest as they can express FAP on both the tumor cells themselves and the associated CAFs [18,65]. While some sarcomas demonstrate high FAP uptake, improvement in staging compared to FDG is not consistent across all subtypes [64,176,177]. Its potential role may be restricted to sarcomas with low FDG avidity but high FAP expression, and importantly, for selecting patients for FAP-targeted radioligand therapy [178,179]. A recent review summarised early clinical data and future perspectives of FAPI in sarcoma, highlighting variable uptake across histologic subtypes and potential theranostic applications [180,181].

#### 4.1.6. Treatment Response Evaluation

Using FAPI for treatment response evaluation is in its early stages, though preliminary studies suggest it can accurately measure response [182,183]. For instance, in giant cell tumors of the bone, FAPI has shown utility in monitoring response to denosumab therapy, with a notable decrease in tracer uptake correlating with histopathological changes [184]. However, treatment-induced fibrosis, inflammation, and necrosis represent potential confounders [63]. External beam radiation therapy, for instance, can induce a fibrotic response that shows FAPI uptake [77]. Similarly, surgery can result in fibrosis visible on FAPI that persists for up to eight months [63]. With the future approval of FAP-targeted therapies, FAPI PET’s role as a biomarker for assessing the therapeutic target will become increasingly critical [185].

### 4.2. Non-Oncologic Applications

As a marker of activated fibroblasts, FAP serves as a promising biomarker for a wide range of inflammatory and fibrosing diseases [64]. This extends to immune-related adverse events, as demonstrated in a case of inflammatory arthritis induced by an anti-programmed death-1 checkpoint inhibitor [186]. The relatively low FAP accumulation in most normal tissues provides a distinct advantage for whole-body imaging of these conditions [30,50]. To aid in accurate image interpretation, Table 4 summarizes common non-malignant causes of FAPI uptake, including physiologic variants and benign conditions that may mimic malignancy.

**Table 4:** Common Non-Malignant Causes of FAPI Uptake and Clinical Correlation.

Category	Examples	Clinical/Imaging Correlation	References
Musculoskeletal/Degenerative	Osteoarthritis, enthesopathy, fractures, degenerative disc disease, periprosthetic uptake	Uptake follows mechanical stress or degenerative changes. CT correlation is critical	Giesel et al. (2019) [30]; Kratochwil et al. (2019) [51]
Inflammatory/Infectious	IgG4-related disease, rheumatoid arthritis, organising pneumonia, tuberculosis, pancreatitis	Uptake may appear diffuse or focal; always correlate with clinical context and CT morphology	Luo et al. (2021) [187]; Chen et al. (2023) [60]
Post-Surgical/Post-Traumatic	Surgical scars, wound healing, post-radiation fibrosis	Uptake often linear or focal; can persist for up to 8 months	Bentestuen et al. (2023) [66]; Luo et al. (2021) [187]; Chen et al. (2023) [60]
Physiologic/Hormonal	Uterine endometrium (premenopausal), lactating breasts, salivary glands	Uptake patterns are symmetric and predictable; correlate with age, hormonal status, and clinical context	Kratochwil et al. (2019) [51]; Giesel et al. (2019) [30]
Inflammatory Bowel Disease (IBD)	Crohn’s disease (active and fibrotic strictures); minimal uptake in ulcerative colitis	FAPI active in Crohn’s strictures but not in ulcerative colitis; enables distinction between inflammatory and fibrotic disease activity; meta-analysis (20 studies, 547 patients) reports detection rate of 89% for Crohn’s inflammation	Abdlkadir et al. (2026) [188]; Luo et al. (2021) [189]
Pancreatic Inflammation	Acute pancreatitis, chronic pancreatitis	FAPI uptake correlates with degree of fibrosis and inflammation; distinguishes pancreatitis from pancreatic malignancy when FDG is ambiguous; enables differential diagnosis in equivocal cases	Abi Ghanem et al. (2024) [190]
Immune-Mediated Inflammatory Diseases (IMIDs)	IgG4-related disease, inflammatory bowel disease (Crohn’s disease), interstitial lung disease, lupus nephritis, renal fibrosi	FAPI detects fibrosis while FDG detects inflammation; enables distinction of inflammatory vs. fibrotic disease activity; guides anti-inflammatory vs. anti-fibrotic therapy selection	Lartey et al. (2025) [191]
Hepatic Fibrosis & Cirrhosis	Liver cirrhosis, advanced hepatic fibrosis	Intense diffuse FAPI uptake reflects activated hepatic fibroblasts; enables non-invasive assessment of cirrhosis severity	Tatar et al. (2023) [192]
Skeletal Muscle & Soft Tissue Injury	Acute muscle injury, chronic muscle injury, myositis, inflammatory myopathies	FAPI uptake correlates with muscle remodeling and fibroblast activation during healing; enables assessment of injury severity and monitoring of repair progression; useful for sports medicine injuries and post-traumatic recovery	Wang et al. (2024) [193]

**Figure 4:**
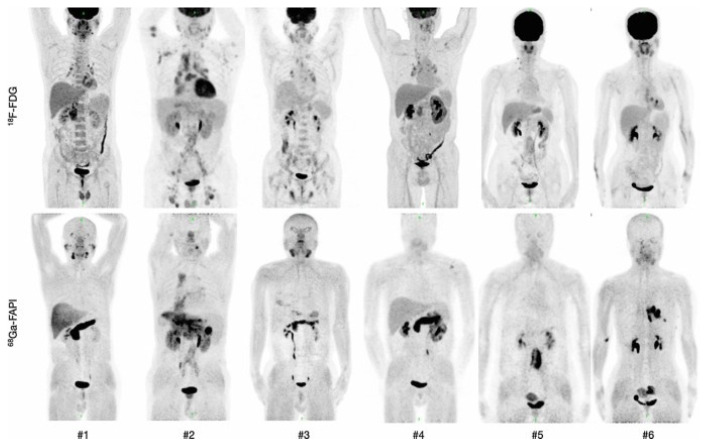
Comparison of FAPI PET/CT and FDG PET/CT in six patients with IgG4-related disease. The FAPI images (lower panels) demonstrate clear uptake in fibrotic and inflammatory lesions—pancreas 1,2,3,4), bile duct/liver (1,2,3), retroperitoneum (5), lung/pleura (6), and salivary glands (1.3)—often exceeding FDG detection sensitivity. From Luo et al. [187].

- Cardiovascular Disease: Unlike FDG, which has diet-dependent variable myocardial uptake, FAP ligands show very low activity in normal myocardium and the cardiac blood pool [61]. This allows FAPI to detect fibroblast activation and cardiac remodeling following acute myocardial infarction [194,195,196]. The fundamental biological validation for this application was provided by Tillmanns et al., who showed that FAPα expression specifically identifies activated fibroblasts after myocardial infarction [197]. Reinforcing this application, further work utilizing FAPI PET/MR has highlighted the potential role of FAPI imaging in predicting the extent of left ventricular remodeling after an acute myocardial infarction [198]. The degree of FAP uptake may have a predictive role in the evolution of ventricular dysfunction [199]. Applications are being explored for detecting fibrosis related to chemotherapy, myocarditis, cardiac sarcoidosis, and light-chain cardiac amyloidosis [200,201,202,203,204,205,206]. Furthermore, the potential role of FAPI has been expanded to include the molecular assessment of fibroblast activation in the right heart of patients with pulmonary arterial hypertension [207]. Given this expanding range of uses, the state-of-the-art role of FAPI in assessing cardiovascular fibrosis has become the subject of dedicated clinical reviews [208,209]. Beyond diagnostics, FAPI is emerging as a tool for risk stratification; a notable study found that myocardial activity on FAPI is associated with an increased risk for sudden cardiac death in patients with hypertrophic cardiomyopathy [210]. Recent work with gallium-labeled PET agents, including FAPI tracers, has expanded cardiovascular molecular imaging, enabling precise evaluation of fibroblast activation in post-ischemic remodeling and chronic myocardial injury [211]. A 2025 review discusses how FAPI-targeted imaging is transforming insights into post-ischemic myocardial remodeling [212].
- Pulmonary Fibrosis: Assessment of pulmonary fibrosis is a particularly promising application [213]. FAPI demonstrates increased signal in fibrotic lung tissue compared to radiographically normal lung in conditions like idiopathic pulmonary fibrosis (IPF) and systemic sclerosis-associated interstitial lung disease (SSc-ILD) [58,214,215]. Early data suggest that higher FAP ligand binding correlates with more active and extensive disease [58,214]. Evidence for novel tracers such as FAPI-LM3 for enhanced detection of early pulmonary fibrosis was recently provided [216]. Further studies are needed to determine if FAPI can predict functional outcomes better than high-resolution CT and pulmonary function tests alone [213]. Preclinical studies have also highlighted the diagnostic accuracy and therapeutic response assessment capabilities of FAPI [217,218].
- Inflammatory and Fibrotic Diseases: Preliminary studies have demonstrated increased FAPI signal in IgG4-related disease, cirrhosis, renal injury and fibrosis, inflammatory bowel disease, and rheumatoid arthritis [60,189,219,220,221,222,223]. This has been extended to autoimmune kidney disease, where Yu et al. used FAPI-04 to assess renal tubulointerstitial fibrosis in lupus nephritis [224]. The scope of FAPI’s application in joint diseases may also include degenerative conditions, as Milner et al. found that FAPα is expressed by chondrocytes and is elevated in osteoarthritis [225]. A 2025 study provides important nuance, showing differences in fibroinflammatory activity on FAPI between the two major subtypes of IgG4-related disease [226]. Preclinical data from Chen et al. using small animal PET/CT in rats further supports fibrosis imaging in other organ systems, such as peritoneal fibrosis [227]. Notably, rheumatoid myofibroblast-like synoviocytes have been shown to express FAP, expanding the diagnostic scope of FAPI in inflammatory joint diseases [228]. In Crohn’s disease, FAPI may help distinguish active inflammatory strictures from chronic fibrotic ones, a critical distinction for guiding medical versus surgical therapy [229]. The biological validation for this approach was provided by Rovedatti et al., who confirmed FAP expression in the fibrotic strictures characteristic of Crohn’s disease [230]. Beck et al. demonstrated that combined FAPI and FDG can distinguish inflammatory from fibrotic activity in Crohn’s-related strictures [231]. In rheumatoid arthritis, FAP uptake correlates with disease activity and may provide a tool for monitoring response to therapy [222,232,233]. The high lesion-to-background contrast makes FAPI particularly illustrative in these conditions (Figure 4).

A 2025 systematic review and meta-analysis of FAPI PET in Crohn’s disease (20 studies, 547 patients) reported a pooled detection rate of 89% for active intestinal inflammation, with FAPI uptake observed specifically in Crohn’s strictures but not in ulcerative colitis [188]. This supports FAPI PET as a tool to distinguish inflammatory from fibrotic disease activity—a clinically critical distinction that conventional imaging cannot reliably provide—and may help guide selection between anti-inflammatory and anti-fibrotic therapy.

Joint disorders represent a further non-oncologic application. Beyond rheumatoid arthritis, FAPI PET has been applied to osteoarthritis, enthesopathies, and periprosthetic joint complications, where it can differentiate active fibroblast activation from quiescent degenerative change [234]. Skeletal muscle injury, including inflammatory myopathies and myositis, has likewise been characterized using FAPI PET, exploiting the activated mesenchymal cell response to muscle damage.

FAPI Imaging Targets: In addition to the original work of Zhou et al. demonstrating FAPI uptake correlating with renal fibrosis severity, Conen et al. showed in a clinical cohort that [^68^Ga]Ga-FAPI uptake correlates with the stage of chronic kidney disease, supporting non-invasive monitoring of renal fibroblast activation as a potential biomarker of disease progression [220,221].

Pancreatic Inflammation: FAPI PET/CT has demonstrated utility in discriminating benign pancreatic inflammation from malignancy. Abi Ghanem et al. presented a case where ^18^F-FDG PET/CT showed an avid infiltrative lesion in the pancreatic head that was ambiguous for malignancy or inflammation, while ^68^Ga-FAPI-04 PET/CT demonstrated diffuse uptake in the pancreatic body and tail consistent with pancreatitis. This illustrates FAPI’s ability to distinguish fibroblast-mediated inflammation from neoplastic disease, with potential clinical utility in acute and chronic pancreatitis assessment [190].

Immune-Mediated Inflammatory Diseases (IMIDs): FAPI PET/CT has demonstrated utility in visualizing and quantifying fibrosis across multiple IMIDs, including interstitial lung disease, IgG4-related disease, renal fibrosis, and inflammatory bowel disease. A key advantage is complementary imaging: FAPI targets fibrosis while FDG detects inflammation, enabling distinction of inflammatory from fibrotic disease phases. This dissociation is clinically critical—in IgG4-RD and IBD, FAPI can guide selection between anti-inflammatory and anti-fibrotic therapy [191].

Liver Cirrhosis and Hepatic Fibrosis: FAPI PET/CT has demonstrated utility in visualizing advanced hepatic fibrosis and cirrhosis. Tatar et al. presented cases of liver cirrhosis where ^68^Ga-FAPI-04 PET/CT showed intense diffuse hepatic uptake, reflecting FAP expression on activated hepatic fibroblasts and myofibroblasts mediating the fibrotic transformation. FAPI imaging may enable non-invasive quantification of cirrhosis severity and fibrosis progression [192].

Skeletal Muscle and Soft Tissue Injury: Beyond fibroinflammatory diseases, FAPI PET/CT has demonstrated utility in evaluating skeletal muscle injury and remodeling. In a clinical study of ^68^Ga-FAPI-04 PET/CT, significant tracer uptake was observed in acute and chronic skeletal muscle injury, correlating with muscle remodeling and fibroblast activation during the healing process. The ability to visualize muscle injury severity and monitor progression of muscle repair could enhance the assessment of sports medicine injuries and post-traumatic muscle healing, particularly in cases with delayed or complicated recovery [193].

### 4.3. Biomarker Concept

FAP is actively being explored as a target for various FAP-directed therapies, creating an urgent need for biomarkers that allow the visualisation and quantification of FAP expression in vivo [31,235,236]. Such biomarkers are essential to enable better selection of patients for these novel treatments and for monitoring their response [23]. While FAP-directed radioligand therapy (RLT) remains in early development, several mechanisms of action are in clinical translation, including monoclonal antibodies, antibody–drug conjugates, and chimeric antigen receptor (CAR)-T cell therapy [28,47,62]. Pursuing FAPI PET as a biomarker can provide regulators with evidence of target engagement by correlating imaging findings with immunohistochemistry (IHC) and offers clinicians a tool for patient selection and monitoring [237].

## 5. Technical Standards and Radiopharmaceuticals

Standardized imaging protocols are crucial to ensure reproducibility across institutions [62,63,65]. Physiologic biodistribution is established rapidly, with the highest activity in the renal collecting system and urinary bladder [34]. Any uptake greater than the surrounding background not attributable to physiologic causes is generally considered suspicious for malignancy. A comprehensive interpretation requires correlation with the patient’s complete medical history and morphological imaging [104,238].

Semi-quantitative analysis using the standardized uptake value (SUV) can be a valuable tool, but consistency requires standardized protocols [239]. Unlike FDG, SUV is less influenced by uptake time. Currently, there is no accepted qualitative uptake scale for FAPI.

### 5.1. Digital and Long Axial Field-of-View (LAFOV) PET Systems

Recent technological advances in digital and long-axial-field-of-view (LAFOV) PET scanners have markedly enhanced sensitivity and temporal resolution, directly influencing the quantitative performance of FAPI imaging. Digital detectors based on silicon photomultipliers (SiPMs) provide higher timing resolution and improved signal-to-noise ratio compared with analog systems, enabling lower injected activities or shorter acquisition times without compromising image quality [52,240].

The new generation of LAFOV scanners—with axial coverage exceeding 100 cm—achieves sensitivities up to 10–15 times higher than conventional PET/CT systems. This allows total-body dynamic acquisitions that capture whole-organ kinetics in a single bed position, offering new opportunities for studying FAPI tracer pharmacokinetics, target engagement, and dosimetry in real time. Such quantitative information may help refine kinetic models of fibroblast activation and improve personalized therapy planning [52,240].

### 5.2. Pitfalls and Physiological Variants

A critical aspect of FAPI interpretation is the recognition of non-tumoral uptake, which is seen in various fibrotic and inflammatory tissues [65]. A retrospective study by Qi et al. specifically investigated non-tumoral uptake of FAPI-04, providing a valuable reference for common pitfalls [241]. Musculoskeletal findings are the most common source of benign uptake [62,66,242,243]. Another benign finding that can show increased uptake is neurofibromatosis, as reported in a patient with pleomorphic rhabdomyosarcoma by Wu et al. [244]. Conversely, this avidity around orthopaedic implants is being investigated as a potential diagnostic strength, with preclinical models suggesting FAPI may help differentiate periprosthetic joint infection from aseptic loosening, a common clinical dilemma [245]. Hormone-responsive uptake in the uterus and breasts is also common [127]. Non-oncologic uptake is also reported in IgG4-related disease, wound healing, and liver cirrhosis [182,187,246]. Dynamic and delayed scanning has been proposed for distinguishing benign from malignant uptake but is not yet recommended for routine practice [44,46,239]. Nonetheless, FAPI uptake in benign processes is a recognized confounder, as highlighted by a systematic review [66]. For example, a case report by Tang et al. showed intense FAPI uptake in organizing pneumonia, which mimicked lung cancer [247]. In the abdomen, chronic colitis is another potential mimic of malignancy on FAPI, as shown in a case report by Yang et al., and even benign tumors such as a presacral schwannoma can mimic malignancy on both FDG and FAPI, as demonstrated by Zhu et al. [248,249].

## 6. Future Directions and Clinical Integration

The future of FAPI PET imaging hinges on addressing current limitations, expanding clinical applications, and pursuing regulatory approval. Reader training is critical and an immediate need [63]. The most important strategy is the execution of well-designed, prospective clinical trials focusing on diagnostic accuracy and clinical impact on patient management and outcomes [250,251]. Table 5 summarizes currently active and recently completed prospective FAPI PET trials that address these validation priorities across multiple clinical scenarios.

A landmark milestone was recently achieved with the publication of results from the first prospective, single-arm, interventional phase 2 clinical trial (NCT05160051) evaluating ^68^Ga-FAPI-46 PET/CT across multiple tumor types with histopathological validation [252]. This trial enrolled 155 participants across a broad range of malignancies and demonstrated that ^68^Ga-FAPI-46 achieves positive predictive values of 93–100% across tumor regions, with superior inter-reader reproducibility compared with FDG PET. Concurrently, a phase 3 trial of [^18^F]FAPI-74 PET/CT for gastric and esophageal cancer is ongoing (NCT07217704), representing the next step toward regulatory approval. These prospective data mark a pivotal transition of FAPI PET from observational to interventional-level clinical evidence.

A recent article introduces FAP-RADS version 1.0, a structured reporting and data system designed for FAP-targeted imaging using PET or SPECT [253]. This five-point scale categorizes lesions based on their likelihood of malignancy, independent of the specific FAP tracer used, thereby ensuring broad applicability and standardizing interpretation across institutions.

The integration of Artificial Intelligence (AI) and machine learning offers potential for automated analysis, and radiomics may provide additional quantitative biomarkers [42,182,254]. Radiomics-based texture analysis of FAPI-46, encompassing parameters such as histogram-based intensity metrics and gray-level co-occurrence matrix (GLCM) features, has demonstrated diagnostic performance with AUCs of up to 0.978 when combined with standard PET metrics [255].

The expansion of theragnostic applications is a particularly promising direction. FAP-targeted RLT is rapidly advancing through clinical trials, using isotopes like Lutetium-177 (^177^Lu), Yttrium-90 (^90^Y), and Actinium-225 (^225^Ac) [54,62,256].

A recent systematic review of FAPI-targeted RLT summarizes the mechanistic rationale, first-in-human safety and efficacy data, and the expanding pipeline of phase 1/2 trials anticipated to mature in the near term [185].

Reader training is a critical and immediate need [63]. Standardisation across institutions remains essential [64]. With the future approval of FAP-targeted therapies, FAPI PET’s role as a biomarker for assessing the therapeutic target will become increasingly critical [185].

## 7. Limitations

Despite its promising performance, FAPI PET imaging faces several limitations. Uptake in benign fibrotic and inflammatory processes can mimic malignancy, leading to potential false positives [65,66]. Tracer retention is variable across tumor types, and cancers with low or heterogeneous FAP expression, such as most lymphomas or prostate adenocarcinomas, may show limited utility [50,51]. Current evidence largely derives from small, single-center studies with heterogeneous protocols, which restricts generalizability [63]. Finally, the lack of regulatory approval and standardized training for image interpretation remain major barriers to routine clinical adoption [49,57].

As a scoping review, this work has inherent limitations. The review protocol was not pre-registered, which may introduce potential selection bias. Because this is a narrative synthesis rather than a formal systematic review with meta-analysis, data were not pooled quantitatively, and risk-of-bias assessment of individual studies was not performed. The inclusion of both peer-reviewed original articles and case reports introduces heterogeneity in evidence quality. Despite a comprehensive search, publication bias toward positive results and the rapidly evolving nature of the field mean that some relevant evidence may not have been captured. Future updates should include prospective registration and a formal assessment of study quality.

Unexplained false-positive FAPI uptake has been reported in anatomical locations where the mechanism of fibroblast activation remains incompletely characterized, including skin folds within axillary and gluteal regions and in the vicinity of pancreatic stents [65,66]. Skin fold uptake may relate to local frictional fibroblast activation, while peri-stent uptake likely reflects FAP activation in the surrounding fibrotic reaction. These patterns cannot always be resolved by CT correlation alone and may require delayed imaging, repeat scanning, or careful clinical contextualization to avoid misinterpretation.

False-negative findings represent an equally important limitation. In low-stage, low-grade tumors with quiescent or inactivated fibroblasts within the tumor stroma, FAPI PET may demonstrate partial or complete photopenia [65]. This phenomenon has been reported in select cases of well-differentiated hepatocellular carcinoma and other indolent malignancies, where minimal fibroblast activation results in absent or substantially reduced tracer uptake despite the presence of viable tumor.

## 8. Conclusions

FAPI PET has emerged as a versatile molecular imaging tool bridging oncology and fibroinflammatory disease. Its strong lesion-to-background contrast, favorable safety profile, and broad diagnostic scope underscore its potential as a next-generation tracer complementing [^18^F]FDG. The joint SNMMI/EANM procedure standards, the publication of FAP-RADS 1.0, the first prospective phase 2 clinical trial with histopathological validation, and increasing multicenter data provide a solid foundation for clinical translation.

However, FAPI imaging remains in the early validation phase. Large-scale, prospective trials are necessary to confirm its diagnostic performance, prognostic utility, and cost-effectiveness. Standardized training programs and consensus interpretation frameworks will be critical for harmonized reporting and regulatory clearance. As digital PET technology, artificial intelligence, and radiopharmaceutical innovation converge, FAPI PET has the potential to become an integral component of precision molecular imaging, transforming disease characterization from metabolism-based to stromal-targeted assessment.

## Figures and Tables

**Table 5:** Verified FAPI PET Clinical Trials (Valid Trials Only).

Trial (NCT)	Intervention	Indication	Phase	Status	Primary Objective
**Oncologic Trials**					
NCT07217704	[^18^F]FAPI-74 PET/CT	Gastric and esophageal cancer	3	Recruiting	Diagnostic accuracy vs. FDG PET; histopathology-confirmed primary endpoint
NCT05263700	^68^Ga-FAPI-46 PET/CT	Cancer of unknown primary (CUP)	2	Recruiting	Detection rate of primary tumor compared with FDG PET
NCT05160051	^68^Ga-FAPI-46 PET/CT	Multiple solid tumors	2	Completed	Histopathology-validated diagnostic accuracy across tumor regions
Non-Oncologic Trials					
NCT07273188	^68^Ga-FAPI-46 PET/CT	Crohn’s disease—fibrostenosis	Early Phase 1	Recruiting	Differentiation of inflammatory vs. fibrotic strictures; early fibrosis detection
NCT04502303	^18^F-FDG and ^68^Ga-FAPI PET/CT	Crohn’s disease	Phase 2	Unknown	Inflammatory vs. fibrotic nature of intestinal strictures
NCT06945549	^18^F-FAPI PET	Inflammatory bowel disease (IBD)	—	Recruiting	Disease activity quantification; intestinal lesion evaluation
Radioligand Therapy Trials					
NCT04939610 (LuMIERE)	^177^Lu-FAP-2286 RLT	Advanced solid tumors (theranostic)	1/2	Recruiting	Safety, tolerability, dosimetry, and preliminary efficacy of FAP-targeted RLT
NCT05400967	^177^Lu-EB-FAPI RLT	Advanced/metastatic solid tumors	Early Phase 1	Recruiting	Safety and dosimetry of 177Lu-EB-FAPI (Evans Blue-modified) RLT

## Data Availability

No new data created.

## Abbreviations

The following abbreviations are used in this manuscript:

AI	Artificial intelligence
AUC	Area under the curve
CAF	Cancer-associated fibroblast
CT	Computed tomography
DPP4	Dipeptidyl peptidase IV
EANM	European Association of Nuclear Medicine
EMT	Epithelial–mesenchymal transition
FAP	Fibroblast activation protein
FAPI	Fibroblast activation protein inhibitor
FAP-RADS	Fibroblast Activation Protein Reporting and Data System
FDG	Fluorodeoxyglucose
HCC	Hepatocellular carcinoma
HNSCC	Head and neck squamous cell carcinoma
ICC	Intrahepatic cholangiocarcinoma
LAFOV	Long axial field-of-view
MRI	Magnetic resonance imaging
NSCLC	Non-small cell lung cancer
PDAC	Pancreatic ductal adenocarcinoma
PET	Positron emission tomography
PRISMA-ScR	Preferred Reporting Items for Systematic Reviews and Meta-Analyses Extension for Scoping Reviews
QA	Quality assurance
QC	Quality control
RLT	Radioligand therapy
SiPM	Silicon photomultiplier
SNMMI	Society of Nuclear Medicine and Molecular Imaging
SPECT	Single photon emission computed tomography
SUV	Standardized uptake value
SUVmax	Maximum standardised uptake value
TBR	Tumor-to-background ratio
TME	Tumor microenvironment
